# A Novel Ultra-Broadband Polarization Filter Based on a Microstructured Optical Fiber with a Gold-Coated Air Hole

**DOI:** 10.3390/mi11090816

**Published:** 2020-08-28

**Authors:** Chao Wang, Yajing Zhang, Zheng Wu, Qian Wang, Guoxu Zhang, Yiyang Zhang, Linghong Jiang

**Affiliations:** 1 Intelligence and Information Engineering College, Tangshan University, Tangshan 063000, China; tzhangyajing@163.com (Y.Z.); goldenfox521@163.com (Z.W.); mo_shen_2000@163.com (G.Z.); txxtgl@163.com (Y.Z.); no1shajia@163.com (L.J.); 2 Key Lab of Intelligent Data Information Processing and Control of Hebei Province, Tangshan University, Tangshan 063000, China; 3 Key Laboratory of Intelligent Motion Control System of Tangshan City, Tangshan University, Tangshan 063000, China; 4 Lucky Healthcare Company Limited, Baoding 071054, China; 15033500392@163.com

**Keywords:** polarization filter, microstructured optical fiber, mode-coupling characteristic, extinction ratio

## Abstract

In this paper, a pentagonal microstructured optical fiber polarization filter by utilizing a surface plasmon resonance effect is proposed. The characteristics of the mode-coupling and filtering of the filter are studied by making use of the full-vector finite element method. The performance of the filter is greatly affected by the structure parameters. The losses of Y and X polarization of the fiber core are 665.97 and 0.17 dB/cm at 1.55 μm, respectively, and the loss ratio is 3917.47. This shows that the filter has a greater loss ratio. Moreover, both the extinction ratio and tolerance are also researched, which shows that the proposed filter has a wider filtering bandwidth and better fabrication tolerance. The designed filter has an important role in wavelength-division multiplexing (WDM) and coherent optical fiber communication systems.

## 1. Introduction

The porous structure of the microstructured optical fiber (MOF) provides convenience for the filling of functional materials, such as liquid crystal [1,2], liquid [3], magnetic fluids [4,5], semiconductor [6], polymer [7,8], and metal [9,10]. In recent years, MOF devices based on material filling, especially metal-filled or coated, which are performed selectively in the hole of a MOF, have become a hot issue of study. When the light travels through the metal-filled or coated MOF and the frequency of the photon bound to the metal surface and the free electron on the metal surface matches, the energy of the electromagnetic field is converted to the vibrational energy of the free electron. The energy on the metal surface will be increased; that is, the surface plasmon resonance (SPR) effect takes place [11]. The MOFs based on SPR play an important role in sensors [12], splitters [13], and polarization filters [14,15,16,17,18,19,20,21,22,23,24,25,26,27,28,29,30], etc. Polarization effects play a key role in a wide range of polarization-sensitive optical fiber systems. Polarization filters can realize only one polarization light transmission, which can be used in resonant fiber-optic gyroscopes to reduce the effects of various optical noises. Moreover, due to the single polarization transmission, the single polarization filter has become a trend to use as a polarization multiplexer in wavelength-division multiplexing (WDM), a polarization switch in the coherent optical fiber communication system, and their performance is directly related to the quality of data transmission. In particular, the MOF polarization filters with the properties of high loss ratio and broadband filtering are the most important part in WDM and coherent optical fiber communication systems.

Up to now, domestic and foreign scholars have launched extensive research on the MOF filters based on SPR and designed many MOF filters with excellent characteristics. In 2006, Kuhlmey B. T. et al. carried out a numerical simulation on the conductive performance of metal-coated MOF by using the multipole method [14]. In 2008, Lee H. W. et al. selectively filled the gold nanowires into the cladding air holes of a MOF [15]. In 2011, Nagaski et al. indicated that the polarized dependent transmission performance of the fiber was influenced by the different filling format and amount of gold wire [16]. In 2013, Xue et al. studied the filtering performance of a gold-coated and liquid-filled MOF filter [17]. The loss of Y polarization of the core is 508 dB/cm around 1.31 μm. Filling liquid can increase the resonance strength. In 2014, An et al. presented a gold-filled filter of a MOF with a triangular and rectangular structure. The loss of Y polarization is 407 dB/cm and that of X polarization is very low at 1.55 μm [18]. In 2015, Chen et al. proposed a D-shaped gold-coated MOF filter. The multiple resonances was generated, and the ultrabroad bandwidth was realized [19]. In 2016, Dou et al. proposed a rhombic single polarization gold-coated MOF filter. The bandwidth of the filter is 520 nm with the 4 mm fiber length. The losses of Y and X polarization are 630.2 and 36.9 dB/cm around 1.55 μm [20]. In the same year, Chen et al. investigated a MOF filter filling gold and liquid crystal. The losses of Y and X polarization are 446 and 0.8 dB/cm around 1.55 μm [21]. In 2017, Shi et al. proposed a rectangular gold-coated MOF filter. The filtering bandwidth is 150 nm with the 4 mm fiber length. The losses of Y and X polarization are 433.65 and 2.64 dB/cm at 1.55 μm [22]. In the same year, Feng et al. presented a broadband core shift filter of a MOF with a big gold-coated air hole. The loss of Y polarization is 584.57 dB/cm [23]. In 2018, Dou et al. designed an square gold-coated MOF filter. The bandwidth of the filter is 520 nm with the 4 mm fiber length. The losses of Y and X polarization are 1005.5 and 1.5 dB/cm around 1.55 μm [24]. In the same year, Feng et al. designed and analyzed a gold-coated MOF filter. The resonant wavelength locating at 1.55 or 1.31 μm is realized by changing the gold film thickness of 17.75 and 33.8 nm [25]. Guo et al. designed a broadband single-polarization gold-coated filter of D-shaped MOF with a micro-opening. The bandwidth of the filter is 480 nm with the 1 mm fiber length. The losses of Y and X polarization are 376.31 and 0.17 dB/cm around 1.55 μm [26]. In 2019, Qu et al. designed a V-shaped gold-coated MOF filter. The realized filtering bandwidth is only 138 nm with the 4 mm fiber length. The losses of Y and X polarization are 689.04 and 8.58 dB/cm around 1.55 μm [27]. In the same year, Wang et al. proposed a rectangular gold-coated MOF filter realizing the simultaneous polarization filtering at 1.31 and 1.55 μm. The loss of Y polarization is 251.5 and 375.3 dB/cm for 1.31 and 1.55 μm, respectively [28]. Liu et al. developed a bimetal-coated and liquid-filled MOF filter, which can simultaneously filter the Y and X polarization modes at 1.31 and 1.55 μm, and the unwanted losses are 544.3 and 147.3 dB/cm, respectively [29]. In 2020, Wang et al. proposed a pentagonal gold-coated MOF filter. The losses of Y and X polarization are 1751.05 and 0.22 dB/cm around 1.55 μm [30]. However, according to the current reports, the polarization loss ratio is still not very high, and the realized wide-filtering bandwidth also needs longer fiber length, which are the two factors limiting the performance and application of the polarization filter; that is, the quality of data transmission in WDM communication system.

A pentagonal polarization MOF filter with a gold-coated air hole has been designed and by taking advantage of the full-vector finite element method (FEM), the filtering characteristic has been analyzed. Compared with the loss of X polarization, Y polarization has an even greater loss, which also makes the filter have a higher loss ratio. The extinction ratio (ER) is −66.58 dB with the 1 mm fiber length. The realized filtering bandwidth with ER less than −20 dB is 140 nm; that is, the filtering wavelength range is from 1.49 to 1.63 μm. In addition, the proposed pentagonal polarization MOF filter has a better fabrication tolerance.

## 2. Structure of MOF Filter

**Figure 1:**
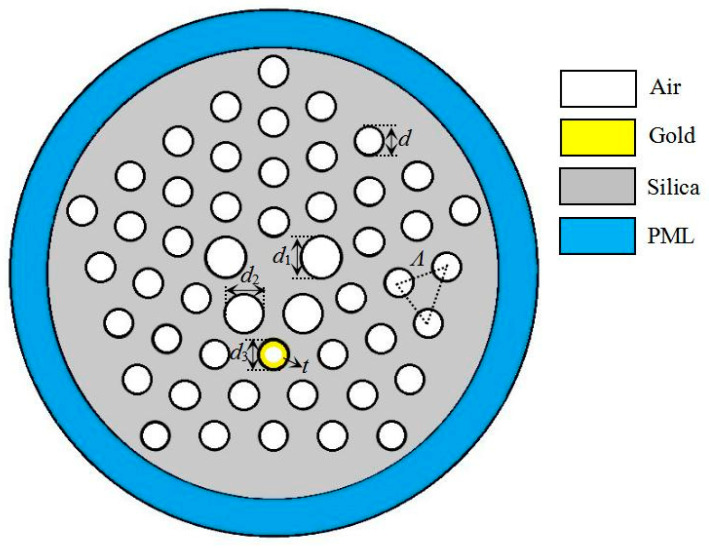
Structure of the microstructured optical fiber (MOF) filter.

Figure 1 displays the structure of the microstructured optical fiber (MOF) polarization filter. The cladding is arranged in pentagonal structure with four layer air holes. *Ʌ* = 2 μm is the distance between two adjacent holes. The hole with *d*_1_ is on the left and right side of the core. The two holes with *d*_2_ are under the core. The hole with *d*_3_ is coated with gold, and the thickness of the gold film is denoted by *t*. The other holes are the same with *d* = 1.2 μm.

In the process of numerical simulation, the silica is used as a substrate material, and its dispersion is expressed by the Sellmeier equation [31]. According to the current reports relating to the MOF filters based on SPR, generally, gold, silver, copper, and aluminum are widely used as the plasmonic material. Gold is chemically stable, and it also shows a larger resonance peak. Therefore, gold is the most commonly used as an excitation plasmonic material. The dielectric constant of gold is described by the Drude–Lorentz model [32], and the refractive index of air is 1. Set the outer layer of the MOF to a perfectly matched layer and scattering boundary condition, which can absorb radiation energy and reduce the reflection energy.

The confinement loss (CL) of the fiber is described as follows [33]:

(1) α(x,y)=8.686×2πλ×Im(neff)×104

Here, Im(*n*_eff_) and λ is the imaginary part of effective refractive index (*n*_eff_) of the fiber core and the light wavelength, respectively. The unit of CL and λ is dB/cm and μm. The size and range of the loss can reflect the performance of the filter to some extent.

The normalized output power (*P_out_*(*x*, *y*)) of X and Y polarization is calculated as follows [34]:

(2) $$P_{out}\left(x,y\right)=P_{in}\left(x,y\right)\exp\left(-\alpha \left(x,y\right)\left(\frac{\ln10}{10}\right)L\right)$$

Here, *P_in_*(*x*, *y*) is the input power and is normalized to 1; *L* is fiber length.

The performance of the filter is measured by utilizing the ER, and it can be calculated by the ratio of X polarization output power to Y. The calculation expression of ER is as follows [35]:

(3) $$ER=10\log_{10}\frac{P_{out}\left(x\right)}{P_{out}\left(y\right)}$$

## 3. Numerical Results and Analysis

### 3.1. Dispersion Relation

**Figure 2:**
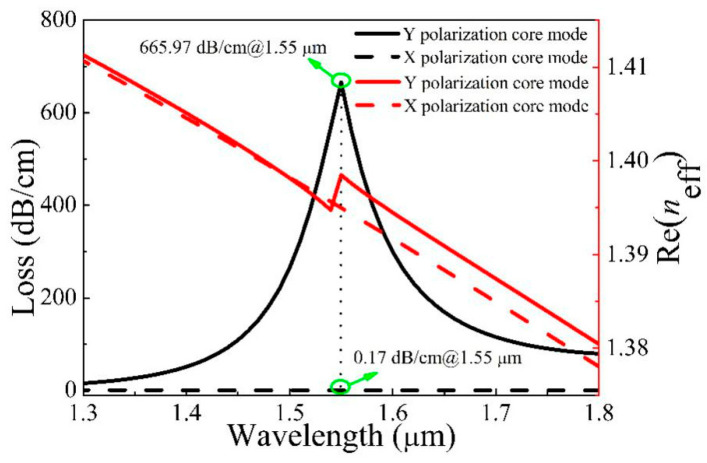
Losses and the real part of the *n*_eff_ (Re(*n*_eff_)) of X and Y polarization of the core change with wavelength.

The structural parameters are optimized by genetic algorithm, and the optimal parameters of microstructured optical fiber (MOF) filter are obtained with *d*_1_ = 1.7 μm, *d*_2_ = 1.6 μm, *d*_3_ = 1.75 μm, and *t* = 31.3 nm. Figure 2 displays the relationship between the loss (left Y axis) and Re(*n*_eff_) (right Y axis) of X and Y polarization of the core. From Figure 2, the Re(*n*_eff_) decreases with increasing wavelength; however, the Y polarization Re(*n*_eff_) curve of the core has an inflection point around 1.55 μm. Meanwhile, the loss of Y polarization increases and then decreases with the increase of wavelength. The maximum occurs at 1.55 μm, and the value is 665.97 dB/cm, which also indicates that the core and surface plasma polarization (SPP) mode is coupled in Y polarization. For X polarization, the change of the loss value is not obvious with increasing wavelength, and the loss is only 0.17 dB/cm at 1.55 μm. This also shows that there is no obvious coupling between the core and SPP mode. Besides, the loss ratio is 3917.47, which also indicates that the single polarization MOF filter of 1.55 μm wavelength is implemented. The light in Y polarization is consumed due to high loss, and only the light in X polarization travels through the fiber core.

**Figure 3:**
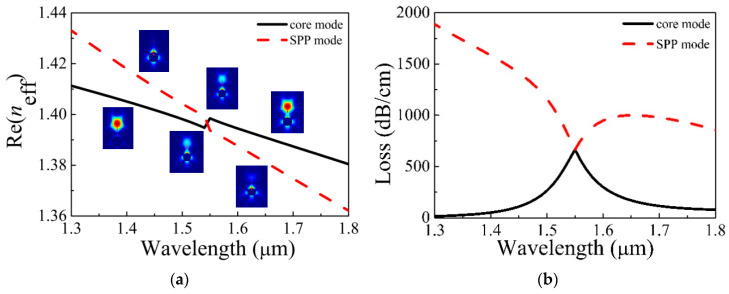
(**a**) Re(*n*_eff_) and (**b**) the losses of the core and second surface plasma polarization (SPP) mode in Y polarization change with wavelength.

Figure 3a displays the change of Re(*n*_eff_) of Y polarization of the core and second SPP mode. The illustration shows the electric field distribution of the core and second SPP mode at different wavelengths. From Figure 3a, at the short wavelength, the field of the core is limited to the core, and that of second SPP is confined to the surface of the gold film. The mode field of the core begins to transfer to the surface of the gold film with increasing wavelength. At the inflection point of the curve—in other words, at 1.55 μm—the core and second SPP mode have the same mode field strength, which is referred to as the resonance wavelength. The energy returns to the core and the surface of the gold film at the long wavelength. Figure 3b shows the losses of Y polarization of the core and second SPP mode. From Figure 3b, the losses of the core and second SPP mode at 1.55 μm are equal when the condition of phase-matching is met, which indicates that the complete coupling has occurred [24].

**Figure 4:**
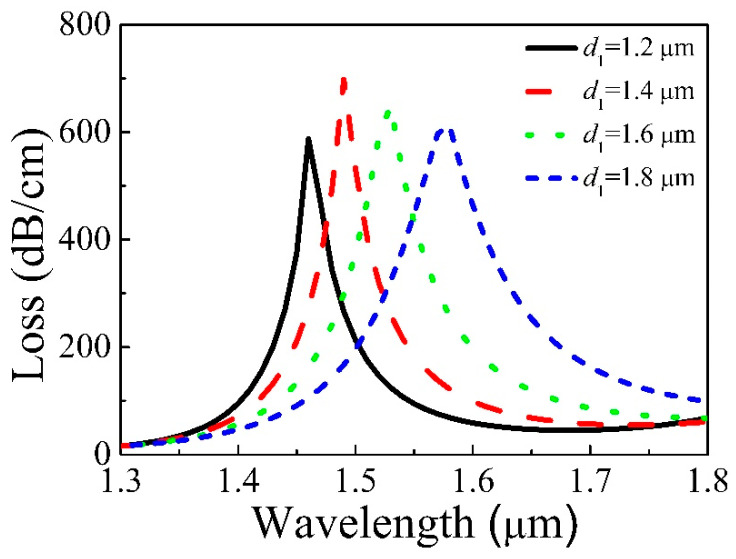
Loss of Y polarization of the core varies with different *d*_1_.

Figure 4 displays the loss of the core varies with *d*_1_ = 1.2, 1.4, 1.6, 1.8 μm, *d*_2_ = 1.6 μm, *d*_3_ = 1.75 μm, and *t* = 31.3 nm. As *d*_1_ increases, the resonance wavelength moves towards the longer wavelength, which can be seen from Figure 4. That is why the Re(*n*_eff_) of the core decreases, and that of second SPP is basically unchanged, which causes the phase matching point to red-shift. The resonance intensity increases and then decreases when *d*_1_ increases. The Re(*n*_eff_) of the core is close to second SPP with increasing *d*_1_, which makes the coupling enhance and more energy of the core be transferred to second SPP. That causes the resonance intensity to increase. However, the further increase of *d*_1_ will cause the core to struggle to limit the light, which will weaken the coupling and make the resonance intensity reduce.

**Figure 5:**
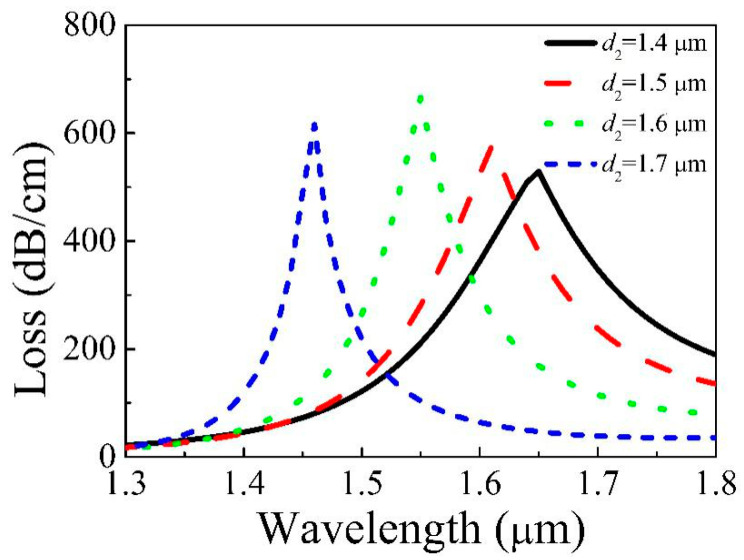
Loss of Y polarization of the core varies with different *d*_2_.

Figure 5 displays the loss of the core varied with *d*_2_ = 1.4, 1.5, 1.6, 1.7 μm, *d*_1_ = 1.7 μm, *d*_3_ = 1.75 μm, and *t* = 31.3 nm. From Figure 5, the resonance wavelength moves towards the shorter wavelength as *d*_2_ increases. The structures around the core and gold film are changed with increasing *d*_2_, which causes the Re(*n*_eff_) of both the core and second SPP to change. The Re(*n*_eff_) of second SPP is greatly reduced, but the variation of the Re(*n*_eff_) of the core is very small, which causes the phase matching point to blue-shift. The resonance intensity augments and then reduces as *d*_2_ increases. That is why increasing *d*_2_ causes the area of the silica bridge between the core and gold film to decrease, which will weaken the coupling.

**Figure 6:**
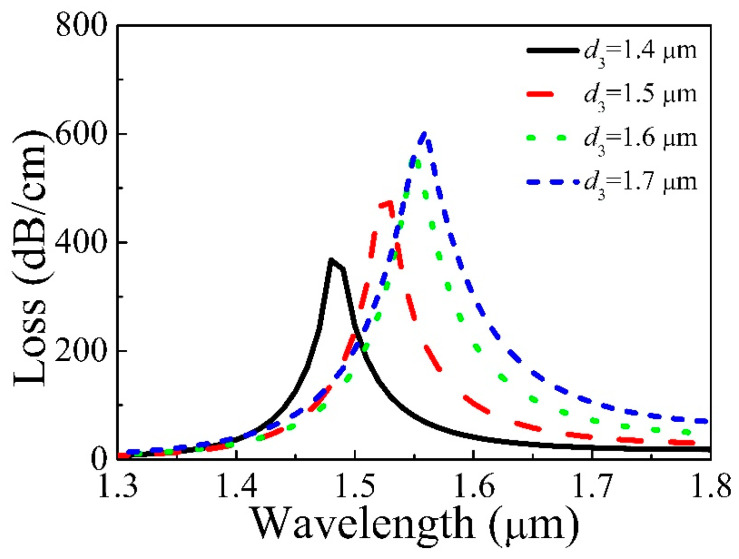
Loss of Y polarization of the core varies with different *d*_3_.

Figure 6 displays the loss of the core varied with *d*_3_ = 1.4, 1.5, 1.6, 1.7 μm, *d*_1_ = 1.7 μm, *d*_2_ = 1.6 μm, and *t* = 31.3 nm. The resonance wavelength moves towards the longer wavelength with increasing *d*_3_, which can be viewed from Figure 6. The structure around the core is not affected with the increase of *d*_3_, but that of the gold film changes. This also makes the Re(*n*_eff_) of the core mode change very little; nevertheless, the change of the Re(*n*_eff_) of second SPP mode is very big, which causes the phase matching point to red-shift. Besides, the distance between the core and gold film decreases with increasing *d*_3_. That will make the coupling strengthen and cause the resonance intensity to rise.

**Figure 7:**
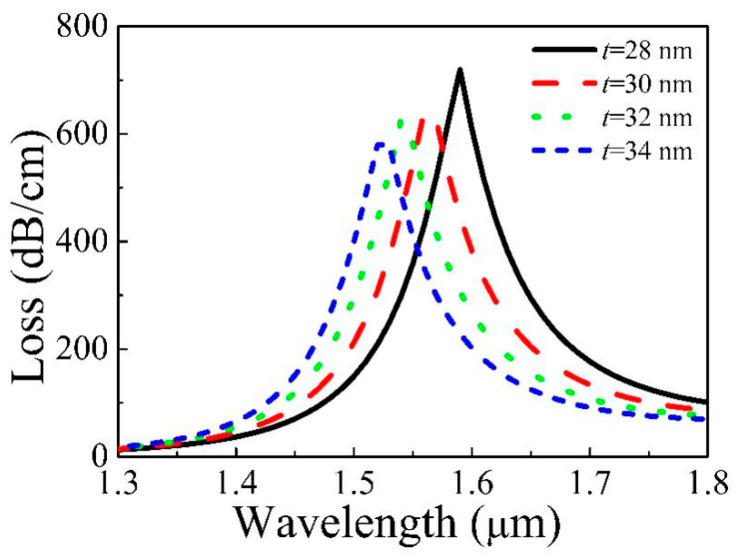
Loss of Y polarization of the core varies with different *t*.

Figure 7 displays the loss of the core varied with *t* = 28, 30, 32, 34 nm, *d*_1_ = 1.7 μm, *d*_2_ = 1.6 μm, and *d*_3_ = 1.75 μm. From Figure 7, the resonance wavelength moves towards the shorter wavelength as *t* increases. Although increasing *t* does not affect the structure around the core, that of the gold film changes. The change of the Re(*n*_eff_) of second SPP has a big reduction, but that of the core changes very little, which causes the phase matching point to blue-shift. Besides, the coupling weakens, so the resonance intensity decreases.

**Figure 8:**
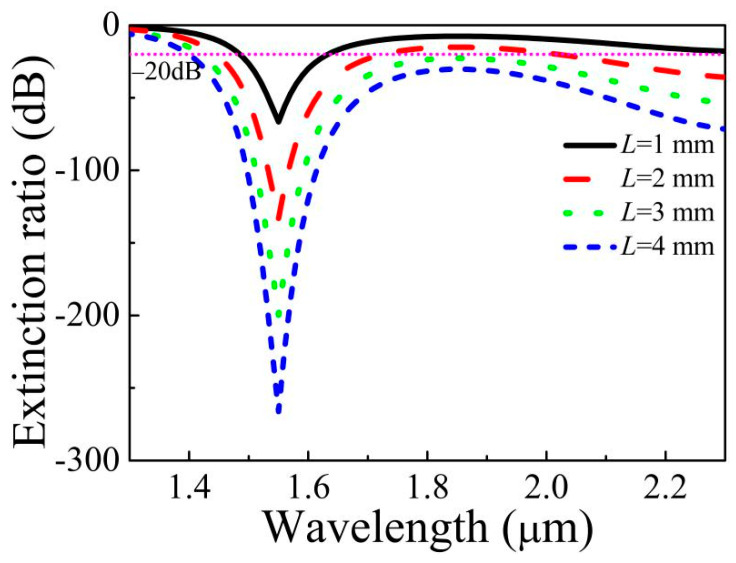
Extinction ratio (ER) varies with *L* when *d*_1_ = 1.70 μm, *d*_2_ = 1.60 μm, *d*_3_ = 1.75 μm and *t* = 31.3 nm.

Figure 8 displays the ER varied with *L* = 1, 2, 3, 4 mm and the structure parameters *d*_1_ = 1.7 μm, *d*_2_ = 1.6 μm, *d*_3_ = 1.75 μm, and *t* = 31.3 nm. Both the ER and the bandwidth with ER less than −20 dB also increase gradually as *L* increases, which can be seen from Figure 8. When *L* is 1, 2, 3, and 4 mm, respectively, the corresponding ER is −66.58 dB, −133.16 dB, −199.74 dB, and −266.32 dB. When *L* is 1 mm, the ER less than −20 dB is the wavelength range from 1.49 to 1.63 μm, and the bandwidth is up to 140 nm. When *L* increases to 4 mm, the realized filtering wavelength range is from 1.40 μm to a longer wavelength, which shows that the designed MOF filter achieves broadband filtering.

### 3.2. Contrast and Discussion

Table 1 displays the comparison results of the designed filter with those previously reported. The high loss ratio and the wide filtering bandwidth are desired parameters in wavelength-division multiplexing (WDM) and coherent optical fiber communication systems. From Table 1, when the fiber length is 4 mm, the loss ratio and filtering bandwidth of the designed filter is 3917.47 and more than 900 nm, respectively. That indicates that the proposed microstructured optical fiber (MOF) filter has a larger loss ratio and wider filtering bandwidth, which is superior to others.

### 3.3. Analysis of the Tolerance

**Figure 9:**
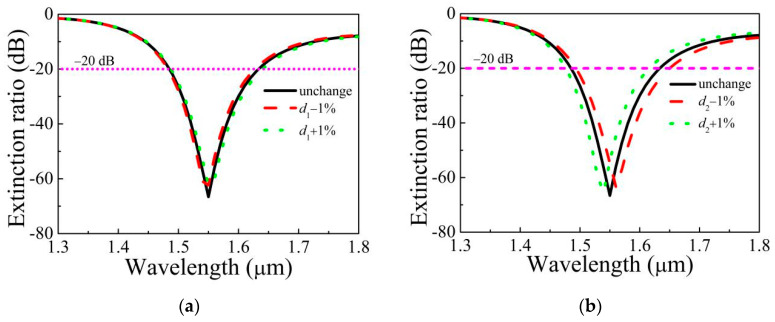

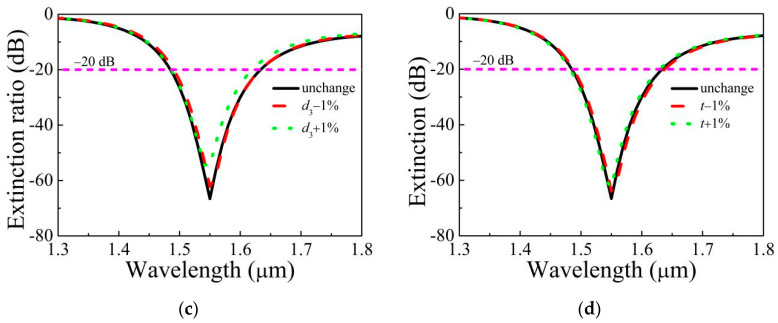
ER varies with wavelength when the structural parameters (**a**) *d*_1_, (**b**) *d*_2_, (**c**) *d*_3_, and (**d**) *t* change ±1%.

The polarization microstructured optical fiber (MOF) filters generally can be made through two steps. Firstly, the designed pentagonal MOF is fabricated by ultrasonic punching [36]. The ultrasonic wave is used to make the required air holes in the silica glass rod to obtain the fiber preform. Then, the preform goes into the fiber drawing tower and pulls to make the pentagonal MOF under the conditions of the suitable pressure and temperature. Secondly, the gold film is plated into the specific air hole by wet chemical deposition or high pressure chemical vapor deposition method [37,38]. It is inevitable that the structure parameters of the fiber changed slightly during fiber drawing. Figure 9 and Table 2 display the ER varied with wavelength when *d*_1_, *d*_2_, *d*_3_, and *t* change ±1%. From Figure 9, the change of *d*_2_ have a slight effect on the ER, while the changes of *d*_1_, *d*_3_, and *t* have little effect on the ER. The ER curves with ±1% deviations of the structural parameters *d*_1_, *d*_2_, *d*_3_, and *t* are almost overlapping. From Table 2, when *d*_1_, *d*_2_, *d*_3_, and *t* change ±1%, the minimum ER and bandwidth with ER less than −20 dB is −58.42 dB and 120 nm with the 100 μm fiber length, respectively. That indicates the proposed filter still has the large loss ratio and wide filtering bandwidth as the tiny deformation occurs, which illustrates that good filtering performance can also be realized. This shows that the MOF filter has good fabrication tolerance.

## 4. Conclusions

A new broadband gold-coated pentagonal microstructured optical fiber (MOF) filter is put forward in this paper. The mode-coupling characteristic and the influence of *d*_1_, *d*_2_, *d*_3_, and *t* on the properties of the filter are researched by using FEM. The resonance wavelength and resonance intensity are greatly influenced by *d*_1_, *d*_2_, *d*_3_, and *t*. When *d*_1_ = 1.7 μm, *d*_2_ = 1.6 μm, *d*_3_ = 1.75 μm, and *t* = 31.3 nm, the core and second SPP are completely coupled in Y polarization direction. The large loss ratio is realized at 1.55 μm, and the value is 3917.47. When *L* is 4 mm, the ER is −266.32 dB, and in the wavelength range greater than 1.40 μm, the ER is less than −20 dB, which also shows that the designed filter has a wider filtering bandwidth. Besides, the designed filter has a better fabrication tolerance. Thus, the filter can be widely used in WDM and coherent optical fiber communication systems.

## Figures and Tables

**Table 1:** Comparison results between the proposed filters and reported filters.

Ref.	Resonance Wavelength (μm)	*α*(*y*) (dB/cm)	*α*(*x*) (dB/cm)	Loss Ratio	Length (mm)	Bandwidth (nm)	Fiber Structure
[20]	1.55	630.2	36.90	17.08	4	520	Rhombic
[22]	1.55	433.65	2.64	164.26	4	150	Rectangular
[24]	1.55	1005.50	1.50	670.33	4	870	Square
[25]	1.31	832.35	N/A	N/A	0.3	150	Square
	1.55	742.44	N/A	N/A	0.3	350	
[27]	1.55	689.04	8.58	80.31	4	138	V-shape
our filter	1.55	665.97	0.17	3917.47	4	>900	Pentagonal

**Table 2:** The impact of the deviation of *d*_1_, *d*_2_, *d*_3_, and *t* from ±1% on the performance of the filter with *L* = 1 mm.

Parameter Change	Resonance Wavelength (μm)	*α*(*y*) (dB/cm)	*α*(*x*) (dB/cm)	Loss Ratio	Min ER (dB)	Bandwidth (ER < −20 dB) (nm)
Unchanged	1.550	665.97	0.17	3917.47	−66.58	140
*d*_1_ − 1%	1.546	668.73	0.16	4179.56	−66.86	130
*d*_1_ + 1%	1.554	660.77	0.18	3670.94	−66.06	140
*d*_2_ − 1%	1.562	646.86	0.20	3234.30	−64.67	140
*d*_2_ + 1%	1.537	681.56	0.14	4868.29	−68.14	130
*d*_3_ − 1%	1.553	650.93	0.18	3616.28	−65.08	130
*d*_3_ + 1%	1.546	672.80	0.16	4205.00	−58.42	120
*t* − 1%	1.553	668.14	0.17	3930.23	−66.80	140
*t* + 1%	1.547	659.17	0.17	3877.47	−65.90	130
